# Synthesis, Characterization, and Cellular Uptake of a Glycylglycine Chelate of Magnesium

**DOI:** 10.1021/acsomega.1c04146

**Published:** 2021-11-30

**Authors:** Derek R. Case, Ren Gonzalez, Jon Zubieta, Robert P. Doyle

**Affiliations:** † 111 College Place, Department of Chemistry, Syracuse University, Syracuse, New York 13244, United States; ‡ Balchem Corporation, 52 Sunrise Park Road, New Hampton, New York 10958, United States

## Abstract

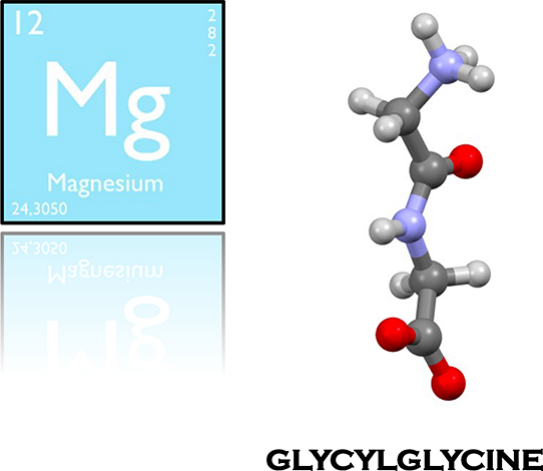

Human chronic latent magnesium deficiency is estimated to impact a substantive portion of the world’s population. A number of magnesium compounds have been developed to combat this deficiency; however, none are ideal due to issues of solubility, absorption, side effects (e.g., laxation) and/or formulation. Here, we describe the pH-dependent synthesis, chemical characterization (inductively coupled plasma and thermal analysis, infrared and nuclear magnetic resonance (1D and 2D) spectroscopies, and electrospray mass spectrometry) and in vitro uptake (in a cell model of the large intestine (CaCo-2 cells)) of a magnesium complex of the glycine dimer (HG_2_). Results demonstrate that the HG_2_ ligand assumes a tridentate coordination mode with an N_2_O donor set and an octahedral coordination sphere completed with coordinated waters. The magnesium:HG_2_ complex exhibits significant solubility and cellular uptake.

## 1 Introduction

Due to insufficient dietary intake, it is now estimated that up to 30% of people living in developed countries may be magnesium-deficient.^1,2^ Magnesium supplements comprise the primary means of palliating the effects of such magnesium deficiency (hypomagnesemia, defined as <0.75 mmol/L in serum), an issue estimated to affect approximately 45% of Americans alone.^3−9^ Although providing a relatively efficacious means of treatment, current magnesium supplements are not ideal as they are often suffering from laxative effects,^10,11^ a lack of water solubility that limits dosing options, incomplete characterization (affecting formulation and dosing), and/or possessing poor gastrointestinal (GI) absorption.^12^ As such, new ligands that may mitigate these issues are of utmost importance.

**Figure 1:**
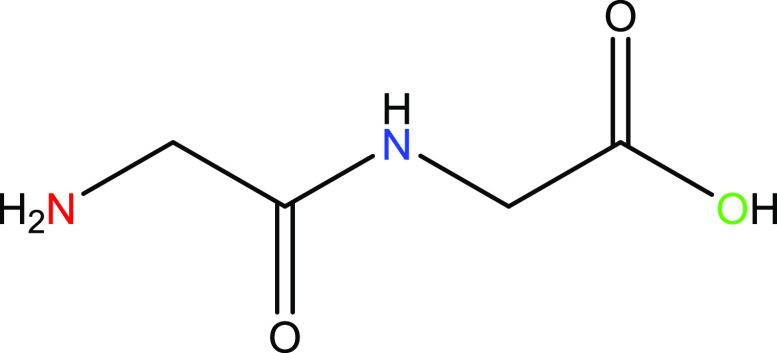
Structure of the dipeptide glycylglycine (HG_2_).

First synthesized in 1901 by Fischer and Fourneau,^13^ glycylglycine (Figure 1; HG_2_)—the simplest canonical peptide—is naturally occurring, having been isolated, along with glycylglycylglycine (triglycine) by Fowden et al. in 1968.^14^ Martell et al. predicted that the HG_2_ ligand would assume a tridentate coordination mode given that no increased complex stability arises upon forming an 8-membered ring.^15^ Given the likely coordination chemistry and its substantial water solubility (22.8 g/100 mL),^16,17^ HG_2_ is an intriguing ligand for magnesium chelation and the subsequent production of a pharmaceutical-grade magnesium supplement. Additionally, previous studies have indicated the substantial uptake of short glycine peptides in man, including HG_2_, providing evidence for the efficacious treatment of deficiencies utilizing ligands of this type.^18^

To this end, the work herein describes the synthesis and full characterization of an HG_2_ chelate of magnesium (**1**) that builds upon and finally completes the initial 1955–1957 work of Martell et al.^15,19^ Complete solution- and solid-state characterization and in vitro uptake of **1** in a standard cell model of the large intestine (CaCo-2), is described.

## 2 Experimental Section

### 2.1 Materials

Magnesium oxide (99.99% metal basis) was purchased from Fisher Scientific (Waltham, MA, USA). HG_2_ (Gly–Gly, ≥99%), MgCl_2_ (BioReagent, ≥97.0%), citric acid (ACS Reagent, ≥99.5%), D_2_O, and DMSO-*d*_6_ NMR solvents, ethanol, and potassium bromide (KBr) for FT-IR analysis were purchased through Sigma-Aldrich (St. Louis, MO, USA). DI water was obtained in-house. Magnesium uptake colorimetric assay kits (catalog #385-100; includes magnesium enzyme mix, assay developer, and magnesium assay buffer) were purchased from BioVision (Milpitas, CA, USA). Stock solutions for magnesium uptake assays were made in-house, with magnesium bisglycinate (MgBG) provided by Balchem Corp (New Hampton, NY, USA) and confirmed for purity in-house by ^1^H NMR and magnesium triglycine (MgG_3_) provided in-house. CaCo-2 (HTB-37) cells and Dulbecco’s modified Eagle medium (30–2002) were purchased from ATCC (Manassas, VA, USA). Penicillin streptomycin (10,000 U/mL), fetal bovine serum (FBS), and trypsin/EDTA (0.25%) were purchased from Gibco (Waltham, MA, USA). White clear-bottomed 96-well Armadillo assay plates (Catalog #AB2396) were purchased from ThermoFisher (Waltham, MA, USA).

### 2.2 Methods

Electrospray ionization mass spectrometry was carried out on a Shimadzu 8040 LC-MS/MS with samples analyzed utilizing a solvent system of H_2_O/MeOH/0.1% TFA at a flow rate of 0.2 mL/min over a 1.5 min time frame and evaluated from 0–600 *m*/*z*. 1D and 2D NMR were conducted on a Bruker Avance III HD 400 MHz instrument. FT-IR was carried out on a Nicolet infrared spectrophotometer. Thermogravimetric analysis (TGA) was carried out on a TA Instrument Q500 from 20–800 °C with sample weights of 5–10 mg. ICP was conducted by Intertek Pharmaceutical Services (Whitehouse, NJ, US). Uptake of magnesium in CaCo-2 cells was determined on a Molecular Devices FlexStation 3 (Molecular Devices). Cellular uptake data was plotted using Prism 8 graphing software.

### 2.3 Synthesis of Magnesium Glycylglycine (1)

**Scheme 1:**
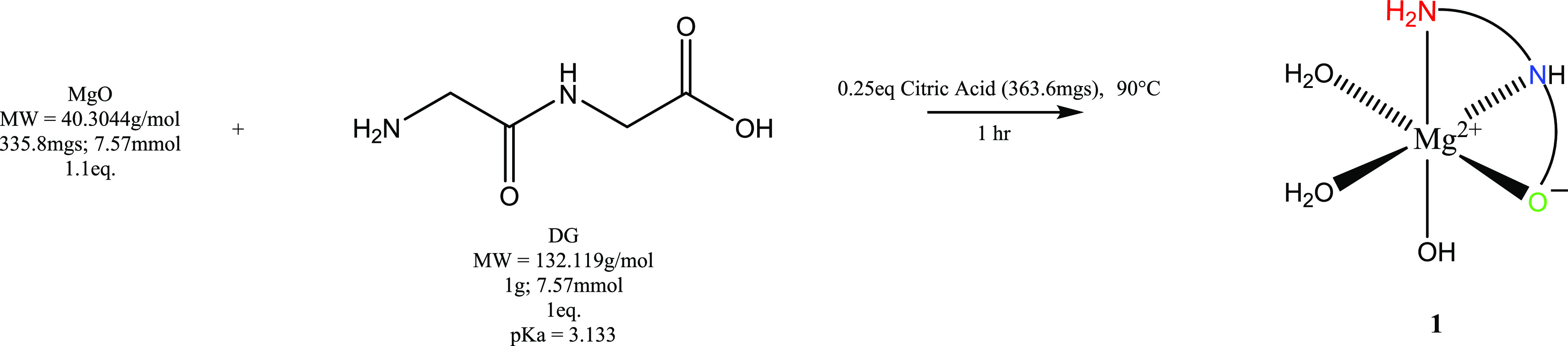
Synthetic Approach to the Synthesis of **1**

HG_2_ (1.02 g, 7.57 mmol) was dissolved in DI H_2_O (∼20 mL) in a 50 mL round-bottom flask, with constant heating at 90 °C and stirring. A separate solution of magnesium oxide (MgO; 0.336 g, 8.33 mmol) was taken up in DI H_2_O (∼20 mL), with an addition of citric acid (CA; 0.364 g, 2.08 mmol (0.25 equiv)), constantly stirred and heated to 90 °C. The MgO/CA solution was added to the HG_2_ solution—upon addition, the combined solution turned an opaque white and was observed as translucent white/clear after about 10 min and up until reaction completion. The reaction was left to run for 1 h at 90 °C. The reaction was cooled to room temperature, centrifuged to pellet any remaining solid, and the supernatant was filtered through a 40 μm filter. The pH of the solution was noted as 10.2. The solution was concentrated in vacuo to approximately 3 mL, and the solid was precipitated with anhydrous ethanol. Centrifugation was employed to pellet the solid, and the ethanol was decanted off. The solid was triturated with diethyl ether ad libitum and centrifuged. The ether was decanted off. To ensure that no trace solvent remained, the solid was reconstituted in DI H_2_O (∼50 mL), flash-frozen, and dried in vacuo on a lyophilizer. The dried material was collected and massed to 1.28 g. Yield was found to be 74% relative to magnesium and 82.6% pure with a 17.3% impurity attributed to magnesium citrate. **1**: ^1^H NMR (D_2_O, 400 MHz): δ 3.76 (s, 2H, H_2_), 3.38 (s, 2H, H_1_). ESMS *m*/*z*: [Mg (G_2_) + H^+^]^+^ calcd for Mg(C_4_H_7_N_2_O_3_) 155.4; found 155, [**1** + H^+^]^1+^ calcd for [MgC_4_H_12_N_2_O_6_ + H^+^]^1+^ 209.5; found 210 (Figure S1). (Mg(C_4_H_7_N_2_O_3_)(H_2_O)(OH)): By ICP calcd: Mg, 8.66%; N, 9.99%; found: Mg, 8.78%; N, 9.89%; calcd ratio Mg:N = 0.87, found ratio Mg:N = 0.89 (Scheme 1).

### 2.4 Culturing of CaCo-2 Cells

CaCo-2 cells were cultured from liquid N_2_ frozen stocks and rapidly thawed to RT using a water bath at 37 °C; cryopreservation media was removed with a micropipette after cells were pelleted via centrifugation for 5 min at 125 g. Cells were resuspended in 1 mL of room temperature Dulbecco’s modified Eagle medium (DMEM) and cultured in 14 mL of DMEM (total volume of 15 mL) with a seeding density of 3.6 × 10^4^ cells/cm^2^ (CaCo-2) in a T-75 cm^2^ culture flask and left to grow in an incubator at 37 °C and 5% CO_2_. Cells were subcultured at 90% confluency, and subculturing occurred in a minimum of five times before use in uptake assays. When cultures reached 90% confluency, media was removed and 3 mL of trypsin/EDTA was added; the trypsinized culture flask was placed back in the incubator for ∼10 min to detach cells. Once detached, cells were confirmed under a microscope, the culture flask was rinsed with 6 mL of fresh media to neutralize the trypsin/EDTA, and cells for uptake assays were then counted utilizing a DeNovix CellDrop. Once counted, cells were pelleted down via centrifugation at 125 g for 10 min, and the supernatant was removed via pipetting. Cells were resuspended in 5 mL of room temperature magnesium assay buffer for cellular uptake assay.

### 2.5 Determination of Magnesium Uptake in CaCo-2 Cells

A colorimetric magnesium uptake assay kit for use with a 96-well plate was purchased from BioVision (Milpitas, CA, US). Sample solutions for use with the kit were prepared in house utilizing magnesium-/calcium-free Hank’s balanced salt Solution (HBSS). The samples tested were magnesium chloride hexahydrate (MgCl_2_**×** 6 H_2_O), **1**, MgBG, and MgG_3_. The kit provided standard for linearity confirmation consists of a 150 nm/μL stock; as such, MgCl_2_**×** 6 H_2_O, utilized as an internal standard, was prepared at this concentration containing 17.93 mM Mg^2+^. All samples were prepared to contain the same amount of Mg^2+^ so as to evaluate magnesium uptake in a relative fashion. DMEM was removed from the plated cells, and cells were subsequently washed three times with HBSS in 100 μL volume. All samples were administered at 150 μL/well as triplicate independent dilutions. Cells were treated for 1–2 h at 37 °C and 5% CO_2_. After incubating, the sample volume was removed from each well and the cells were again washed three times with HBSS. Cells were lysed utilizing 200 μL of kit assay buffer, the post-lysis volume was collected, and each sample was centrifuged at 14,000*g* for 10 min. The resulting supernatants were replated in the same order in 50 μL volume. Fifty microliters of kit-provided enzyme/buffer/developer mix was added to each well with a multichannel micropipette, and the plate was allowed to incubate for 40 min at 37 °C. Some wells were left blank for required background subtraction. The kit-provided standard was diluted to 0, 3, 6, 9, 12, and 15 nmol/μl in DI H_2_O and administered and developed in the same volumes as the samples and was used only to determine kit linearity (see Figure S12). Each well was analyzed for endpoint value over nine full-plate scans with triplet scans/well/plate scan (a total of 27 scans per well), and the reported value of each well was the average value of these scans after background subtraction. All samples were analyzed in triplicate. Data was collected at 40 min. Raw data was reduced and plotted as absorbance against magnesium concentration of each well. All assays were repeated in triplicate—error bars were shown in a graph (Figure 9) (SEM: MgCl_2_ = 0.0006, MgBG = 0.0006, 1 = 0.0007, MgG_3_ = 0.001; upper 95% C.I.: MgCl_2_ = 0.004, MgBG = 0.003, 1 = 0.004, MgG_3_ = 0.006; lower 95% C.I.: MgCl_2_ = 0.001, MgBG = 8.98 × 10^–5^, 1 = 1.11 × 10^–5^, MgG_3_ = 0.0005).

## 3 Results and Discussion

### 3.1 Structural Characterization of 1 Via Infrared Spectroscopy

**Figure 2:**
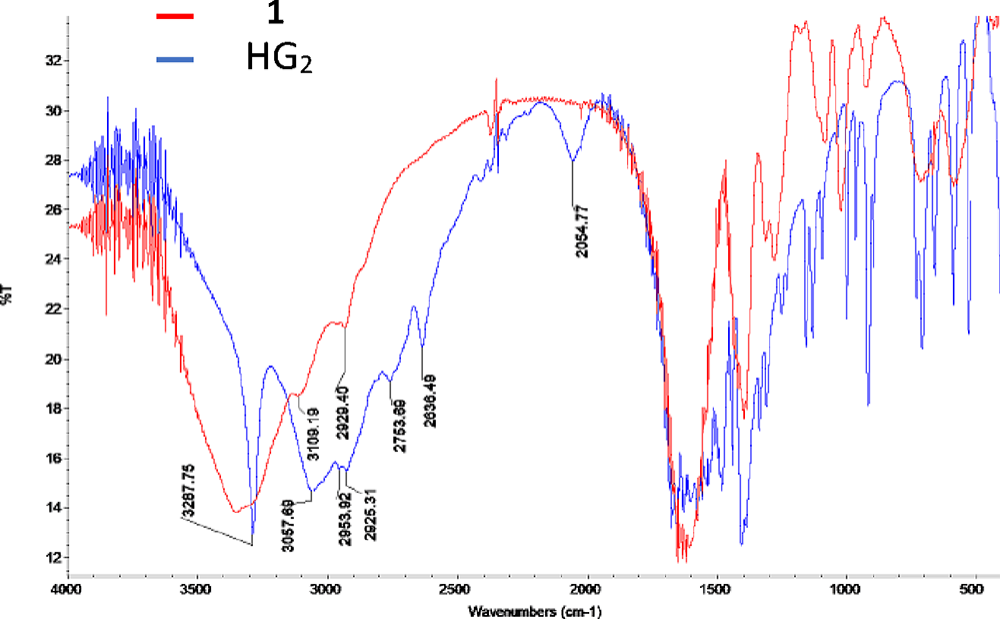
FT-IR spectrum of HG_2_ and 1 in KBr.

The FT-IR of **1** relative to HG_2_ exhibited a substantial change in the frequency region that corresponds specifically to the −OH stretching mode attributed to the carboxylic acid of the HG_2_ ligand at 3287 cm^–1^. HG_2_ exhibited a sharp stretching band in this region that is not observed for **1**, providing support for the deprotonation of the acid (*p*K_a_ = 3.14). Additionally, HG_2_ exhibits a broad signal at 2055 cm^–1^, which is attributed to the terminal amine (−NH_3_^+^),^21^ a signal not observed for **1**. This implicates that both the terminal amine and the terminal acid are in coordination. The FT-IR spectra of HG_2_ and **1** are provided as Figure 2. IR values are provided as Table 1.

**Table 1:** Infrared Spectroscopy Values for HG_2_ and **1**

complex	IR frequency (cm^–1^)	assignment
HG_2_	3287	v(OH), C–OH
	2055	v(−NH_3_^+^)
**1**	3360	v(OH), H_2_O
		v(−NH_3_^+^)

### 3.2 Determining the Chemical Composition of 1 Via ICP and Thermal Analyses

Duplicate independent ICP analyses were conducted on **1** from the same synthetic batch. Acquisition of nitrogen values is complicated by the substantially hygroscopic nature of 1. ICP analysis elucidated the percent magnesium present in the samples and thus provided compositional insight utilizing a Mg:N ratio. The ICP provided values of Mg = 8.78% and N = 9.89% (Figure S11). The values are consistent with a species of composition Mg(G_2_)(H_2_O)(OH) **×** 5 H_2_O. The theoretical nitrogen and magnesium values for this species are Mg = 8.66% and N = 9.99%. Utilizing the Mg:N ratio, the experimental ratio of M:N = 0.89 and is consistent with the theoretical value of Mg:N = 0.87.

**Figure 3:**
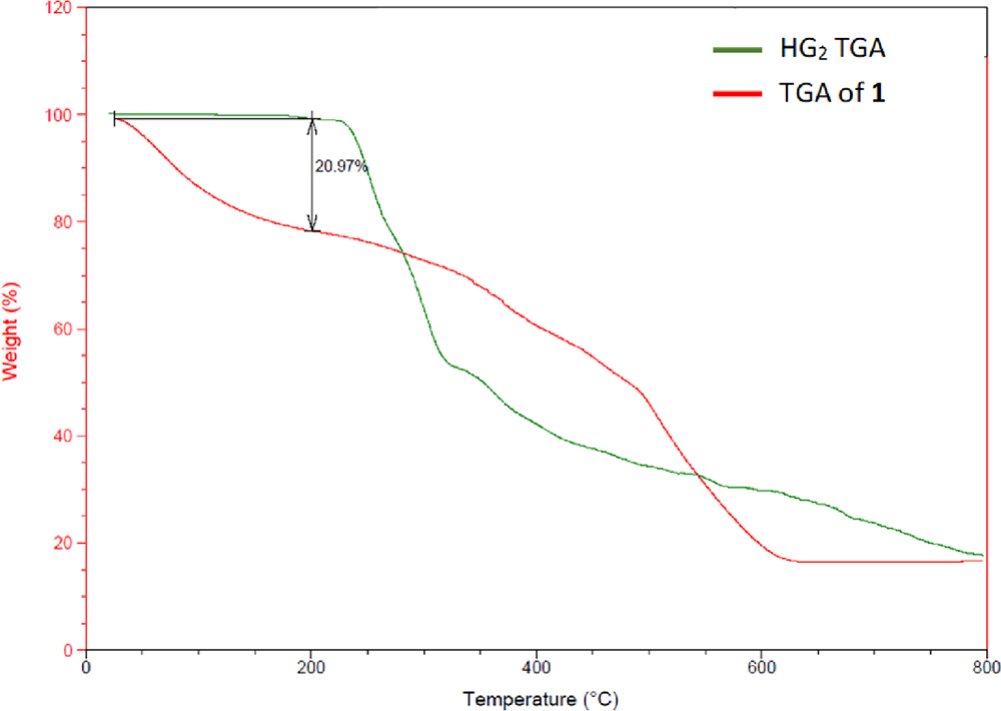
Overlay of HG_2_ TGA (green) and TGA of **1** (red).

TGA of HG_2_ exhibited a single, gradual percent weight decrease onset at 220 °C (inflection point observed at 270 °C), which is consistent with the melting point of HG_2_ at 220 °C. TGA analysis of **1** indicated a gradual decline in percent weight onset from 30 °C until just before 200 °C. The weight change accounts for a loss of 21%, which is consistent with the loss of 2 waters (calculated to 19%) (Figure 3). This result is consistent with the tendency of magnesium to take on water in a rapid fashion,^22,23^ thus suggesting the core magnesium species Mg(G_2_)(H_2_O)(OH) where rapid acquisition of subsequent water molecules is likely.

### 3.3 Structural Characterization of **1** Via 1D and 2D ^1^H/^13^C NMR

**Figure 4:**
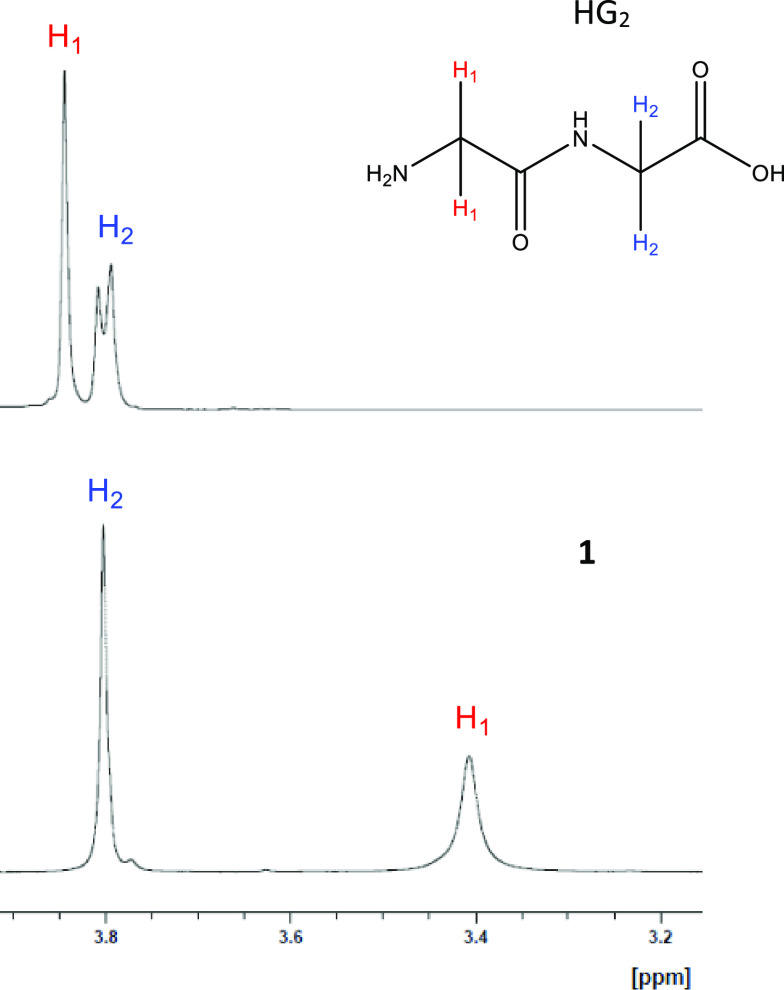
^1^HNMR overlay of HG_2_ (top) and **1** (bottom) conducted in D_2_O. Asterisks indicate peaks attributed to magnesium citrate.

Further support for the coordination mode of **1** was provided in the form of solution-state NMR analysis at equimolar concentration. ^1^H NMR of **1** was conducted relative to the HG_2_ ligand. The ^1^H NMR spectrum of HG_2_ exhibited two proton signals with a combined integration of two (2) (not sure what this 2 means); the observed signals were a sharp singlet at 3.84 ppm (H_1_) and a doublet centered around 3.80 ppm (H_2_; Figure 4; full spectra ^1^H NMR of HG_2_ available as Figure S2). Additionally, the ^1^H NMR **1** exhibited a combined integration of four (4). These observations were consistent with the hypothesized observations. Like the ^1^H NMR spectrum of HG_2_, spectral analysis of **1** exhibited two proton signals with a combined integration of four (4). Unlike HG_2_, the observed proton signals of **1** were both singlets, and each singlet exhibited a substantial upfield shift: H_1_ = 3.38 ppm (Δppm = 0.46) and H_2_ = 3.76 (Δppm = 0.04) (Figure 4; full ^1^H NMR spectrum of **1** is provided as Figure S3). Furthermore, spectral analysis of **1** revealed a quartet between 2.50–2.70 ppm attributed to magnesium citrate of a 1:1 magnesium:citrate composition when compared to a magnesium citrate standard, which is a result of citric acid utilization during synthesis (Figure 4 and Figure S5). Full spectra ^1^H NMR overlay indicating solvent calibration is provided as Figure S4.

As previously reported by Case et al. when analyzing a triglycine chelate of magnesium, the more substantial upfield shift of the proton nearest the terminal amine (H_1_) implicates the participation of this moiety in coordination.^20^ Additionally, coalescing of the H_2_ proton signal for **1** relative to HG_2_ suggests coordination to the carboxylic acid that results in an even distribution of electron density and subsequent lack of observed splitting. Furthermore, the observation of upfield proton shifting coincides with generalized magnesium coordination and may be observed for the α-proton adjacent to the point of coordination and as far-reaching as the γ-proton.^24−27^ Conserved integration of the HG_2_ proton signals is consistent with no formation of new ligand-based product and is consistent with the 1:1 stoichiometric yield of the previously discussed synthesis.

**Figure 5:**
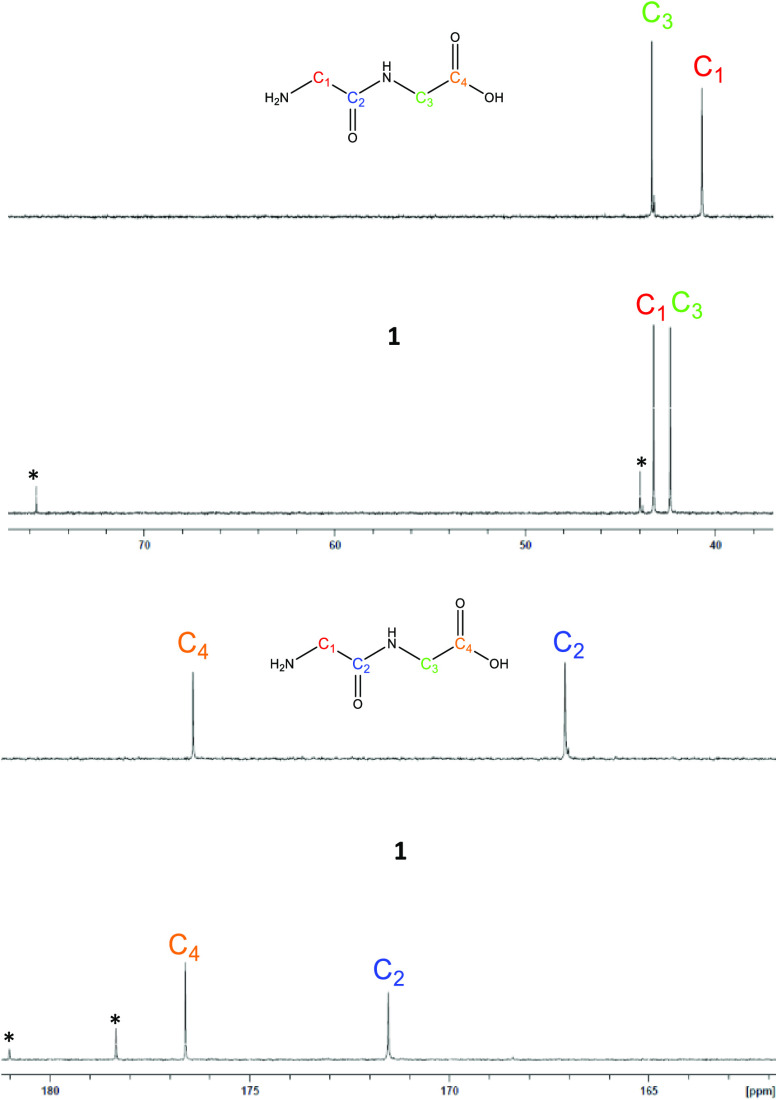
^13^C NMR overlay of HG_2_ and **1** sp^3^-hybridized carbons (top) and HG_2_ and **1** sp^2^-hybridized carbons (bottom) conducted inD_2_O. Asterisks indicate peaks attributed to magnesium citrate.

^13^C NMR was conducted with the aim of confirming the conclusions derived from infrared and ^1^H NMR analyses and is supported as a more sensitive method for determining the mode of magnesium coordination.^26,27^ The ^13^C NMR of **1** was conducted relative to HG_2_. Spectral analysis HG_2_ revealed four carbon signals: two signals between the range of 40–50 ppm attributed to the sp^3^-hybridized R–CH_2_–R carbon moieties (C_1_, 40.7 ppm; C_3_, 43.4 ppm) and two signals between 160–180 ppm attributed to the sp^2^-hybridized R–CO–R moieties (C_2_, 167.1 ppm; C_4_, 176.4 ppm) (Figure 5). ^13^C analysis of **1** exhibited four carbon signals in the same regions as the free HG_2_ ligand. In contrast to the ^13^C spectrum of HG_2_, the signal separation was substantially diminished, which is a result of the downfield shift of the C_1_ (Δppm = 3) and C_2_ (Δppm = 8) signals to 43.2 ppm and 175 ppm, respectively. These observations are consistent with those reported by Drevenṧek et al.^24^ and Chang et al.^27^ in their studies of magnesium testosterone and magnesium ofloxacin/levofloxacin, respectively and are consistent with magnesium coordination in regions adjacent to these moieties. Additionally, in agreement with ^1^H NMR analysis, ^13^C NMR analysis of **1** exhibited four signals attributed to magnesium citrate (Figure 5 and Figures S7 and S8), and no new ligand-based carbon signals were observed, further supporting the formation of **1** as a 1:1 complex.

**Figure 6:**
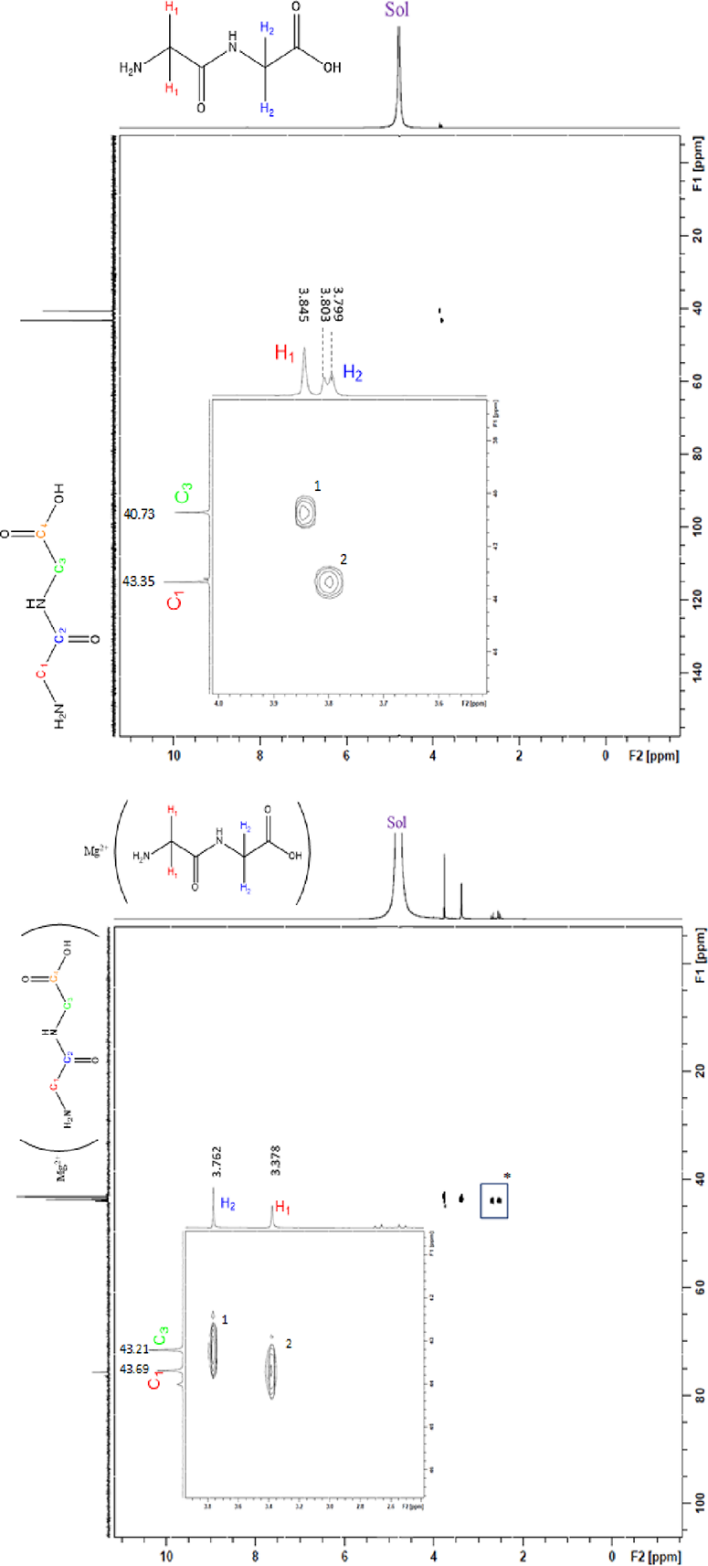
2D HSQC of HG_2_ (top) and HSQC (bottom) of **1** conducted in D_2_O. “Sol” represents the residual HOD peak.

**Figure 7:**
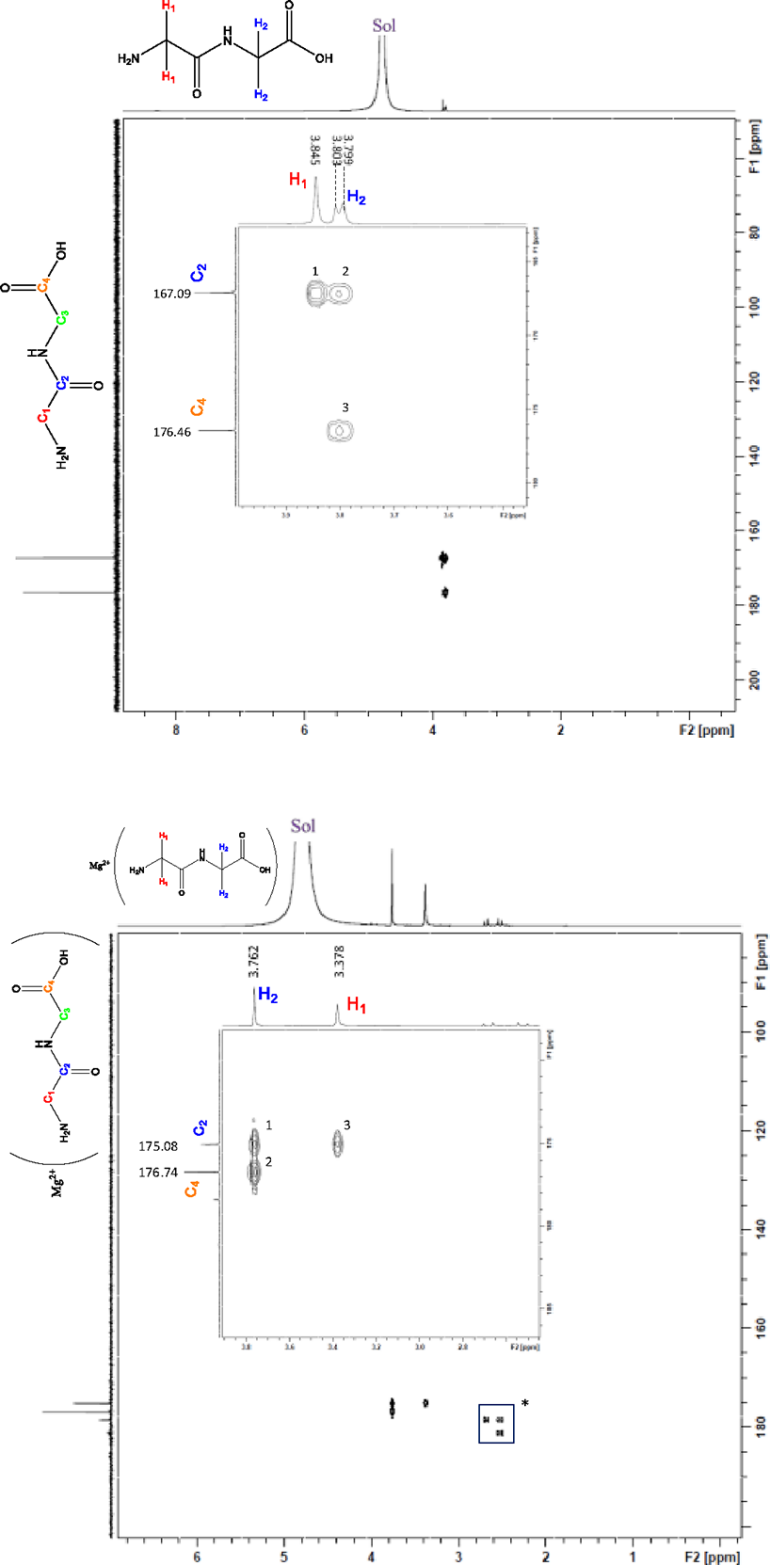
2D HMBC of HG_2_ (top) and HMBC (bottom) of **1** conducted in D_2_O. Asterisks indicate signals attributed to magnesium citrate. “Sol” indicates the residual HOD signal.

Both 2D ^1^H/^13^C heteronuclear single quantum coherence (HSQC), which shows correlations between a defined proton and the carbon a single bond distance away, and heteronuclear multiple-bond correlation (HMBC), which confirms the interaction defined proton and corresponding carbons over multiple bond lengths, confirmed all proton and carbon signal assignments. The HSQC spectrum of HG_2_ (Figure 6) indicated two correlation points: one point attributed to H_1_ and one point attributed to H_2_. The point attributed to H_1_ corresponded to the carbon signal at 40.7 ppm, confirming this to be C_3_, and the point attributed to H_2_ corresponded to the carbon signal at 43.4 ppm, confirming this carbon to be C_1_. The HMBC of HG_2_ (Figure 7) showed three correspondence points of ratio 1:2. The proton signal attributed to H_2_ exhibited two correspondence points. Given that H_1_ is within range of only one carbon, this indicates that the observed correspondence point for H_1_ at 167.1 ppm is attributed to C_2_. Additionally, H_2_ shows two correspondence points. Given that the C_2_ carbon signal corresponds to two protons, this confirms the assignment of the C_2_ proton given its proximity to both protons, and the remaining carbon signal at 176.5 ppm is confirmed as C_4_ given that it is out of range of the H_1_ proton (Figure 7).

HSQC analysis of **1** (Figure 6) indicates two correspondence points like that of the free HG_2_ ligand (Figure 6). In contrast to the HMBC of the free HG_2_ ligand, the ratio of correspondence points observed for **1** is switched (2:1) (Figure 7). Given the observations made for the HMBC of HG_2_, this indicates that the more substantial upfield shift attributed to the H_1_ proton during ^1^H NMR analysis is consistent, with terminal amine participation in coordination. Additionally, the HMBC confirms the H_2_ assignment and implicates the terminal carboxylic acid moiety, which was previously supported by infrared analysis.

A combination of both 1D and 2D ^1^H/^13^C NMR provides insight into the overall coordination mode of the HG_2_ ligand and indicates that the ligand acts as a tridentate chelate that coordinates via an N_2_O donor set. This coordination is achieved via the terminal amine, the backbone amide, and the terminal carboxylic acid. These results are consistent with magnesium chelate ligands assuming higher-order coordination modes that favor entropically stable complexes,^28−30^ as well as previous studies that indicate the tendency of ligands such as HG_2_ and triglycine to act as tri- and tetradentate magnesium chelates.^15,19^ Lastly, all NMR analyses of **1** exhibit minimum amounts of magnesium citrate, confirming an 82.6% purity of **1**.

### 3.4 Overall Discussion on the Structure of **1**

**Figure 8:**
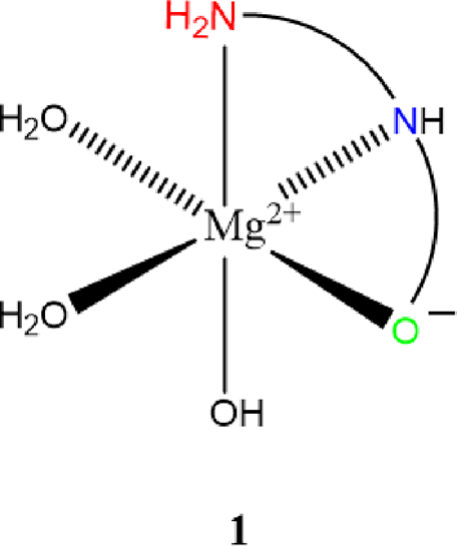
The predicted chemical structure of **1** based on solution- and solid-state analyses**.** Waters of crystallization are omitted for clarity.

The combined solution- and solid-state data is consistent with the structure as shown in Figure 8. The HG_2_ ligand acts as a tridentate magnesium chelate and assumes an N_2_O donor set to form an entropically-favored complex. Additionally, the six-coordinate, octahedral coordination sphere is completed by two waters and one hydroxide. Confirmation of the presence of a hydroxide anion was provided by a series of potentiometric experiments evaluating the conductivity of **1** relative to potassium chloride (KCl; a 1:1 electrolyte). Solutions of both KCl and **1** were prepared at concentrations of 1, 10, and 100 mM, respectively. At 100 mM, data indicated that KCl has a conductivity of 13.11mS and **1** has a conductivity of 5.08 mS (approximately 2.5× less than that of KCl). These data indicate that **1** acts as a 1:1 electrolyte with only partial dissociation, which is consistent with the previously reported MgG_3_.^20^ Conductivity values for both KCl and **1** trend linearly at the given concentration range with *R*^2^_KCl_ = 1.0000 and *R*^2^_**1**_ = 0.9995.

### 3.5 Determining the Water Solubility of 1

The solubility of **1** was determined via triplicate independent analyses and compared to other reported magnesium chelate complexes. Analysis indicated that **1** has a water solubility of approximately 39 g/100 mL. While more soluble than MgO, **1** is less soluble than MgCl_2_ and about one fifth as soluble as MgG_3_, which is previously reported by Case et al. (Table 2).^20^ The water solubility of **1** is attributed in part to the inherent change to a more polar complex given the substantial electropositive character of the divalent magnesium and the dipole alteration generated upon ligand coordination. Additionally, as was the case with MgG_3_,^20^ it is likely that the extensive hydrogen bond network of the HG_2_ ligand is efficacious in generating increased water solubility.

**Table 2:** Water Solubilities of Previously Reported Magnesium Chelate Complexes and Common Magnesium Compounds

complex name	molecular weight (g/mol)	formula	% Mg in compound	solubility (g/100 mL)	ref
MgG_3_	265.5	[Mg(C_6_H_10_N_3_O_4_)(H_2_O)_2_]OH	9.2	169 ± 12.5	(20)
MgCl_2_	95.2	MgCl_2_	25.5	54	(31)
MgO	40.3	MgO	60.3	0.010	(12)
**1**	191.5	Mg(G_2_)(H_2_O)(OH)•*X*H_2_O	11.6	39.40 ± 2.84	this work

### 3.6 In Vitro Cellular Uptake of **1**

**Figure 9:**
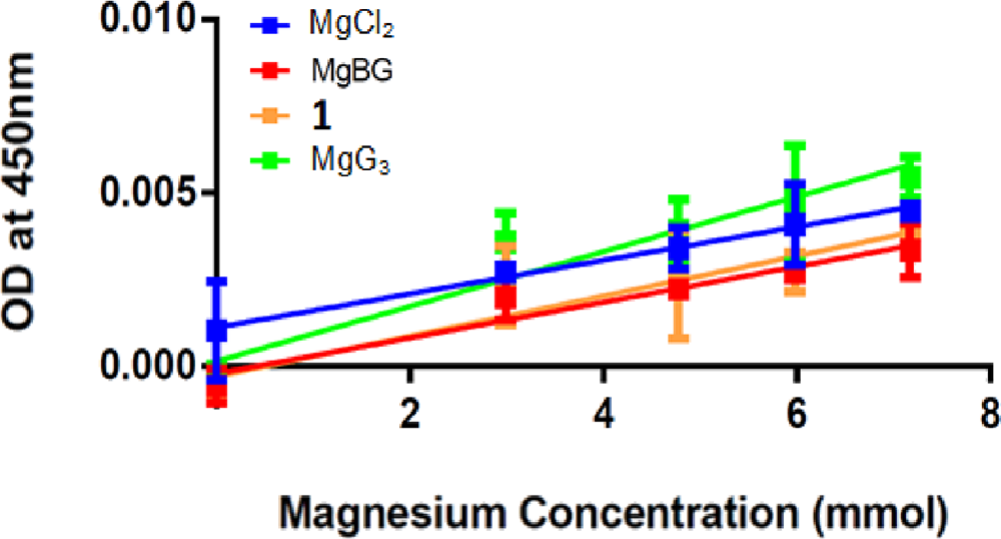
Cellular uptake of **1** (orange) plotted with magnesium bisglycinate (red), magnesium triglycine (green), and MgCl_2_ (blue) in CaCo-2 cells. Slope/*R*^2^ MgCl_2_: 5 × 10^–5^/0.7373. Slope/*R*^2^**1**: 5 × 10^–4^ /0.7231. Slope/*R*^2^ MgBG: 5 × 10^–4^/0.8477. Slope/*R*^2^ MgG_3_: 7 × 10^–4^/0.8019. SEM: MgCl_2_ = 0.0006, MgBG = 0.0006, **1** = 0.0007, MgG_3_ = 0.001; upper 95% C.I.: MgCl_2_ = 0.004, MgBG = 0.003, **1** = 0.004, MgG_3_ = 0.006 ; lower 95% C.I.: MgCl_2_ = 0.001, MgBG = 8.98 × 10–5, **1** = 1.11 × 10–5, MgG_3_ = 0.0005. Kit linearity provided in Figure S12.

Cellular uptake of **1** was evaluated in a lower intestinal colorectal carcinoma (CaCo-2) cell line relative to select previously reported magnesium chelate complexes and MgCl_2_ (Figure 9). Uptake is plotted as a linear regression.

Analysis of cellular uptake data indicates that **1** exhibits uptake less than that of MgCl_2_ and comparable to MgBG. Furthermore, observed uptake of **1** was only about half that of the previously reported MgG_3_ complex.^20^ Uptake increases with increased peptide length from MgBG, **1**, and MgG_3_. This is consistent with the uptake observed in humans for the free mono-, di- and tripeptide glycine ligands observed by Craft et al.^18^ Craft explains that the rate of cellular uptake of the free ligands is directly related to the available mole quantity of the ligand and the corresponding rate of uptake as it pertains to the amount of ligand available.^18^ It is believed that in the case of the uptake of magnesium chelates of glycine-based complexes as reported, Craft’s discussion supports the observable uptake trend. The trend of increasing uptake with a corresponding increase in peptide length is also supported by the findings of Hellier et al., who observed greater intestinal absorption of HG_2_ relative to glycine in human studies.^32^

Additionally, uptake appears to exhibit trending relative to complex solubility. Further in vivo testing is required to confirm this uptake trend at concentrations near complex solution saturation.

## 4 Conclusions

Both solution- and solid-state methods confirm the successful synthesis of a 1:1 magnesium-HG_2_ complex: **1**. Both 1D and 2D ^1^H/^13^C NMR as well as FTIR confirm that the HG_2_ ligand acts as a tridentate chelate and coordinates via an N_2_O donor set utilizing the terminal amine, the backbone amide, and the terminal carboxylic acid. This coordination mode is consistent with the hypothesis previously reported by Martell et al.^15,19^ Additionally, **1** has a predicted distorted octahedral geometry with the remaining three coordination sites not utilized by the HG_2_ ligand, occupied by water and a hydroxide anion for charge balance, which was subsequently confirmed utilizing conductivity. Furthermore, solid-state analyses indicate the propensity of **1** to adsorb water. This is observed in both the ICP and thermal analyses of **1,** exhibiting pentahydrate and tetrahydrate composition, respectively. The in vitro uptake of **1** was comparable to that of the bisglycine chelate MgBG, although both were significantly lower than that of MgCl_2_ or the triglycine chelate MgG_3_, indicating a trend of increasing uptake with increasing peptide length and higher-order coordination (bi-, tri-, and tetradentate). Further in vivo testing is required to confirm the observed in vitro trends.

## Supporting Information Available

Full spectra ^1^H/^13^C NMR of HG_2_/**1**; confirmation of magnesium citrate impurity via ^1^H/^13^C NMR; raw elemental analysis values; cellular uptake of **1** relative to percent composition of magnesium (PDF)

## Notes

R.P.D. sits on the scientific advisory board of Balchem Corp. (New Hampton, NY, United States).

## Abbreviations

MgBG: magnesium bisglycinate
MgG_3_: magnesium triglycine
CaCo-2: colorectal carcinoma cells differentiated into human intestinal epithelial cells
MgCl_2_: magnesium chloride
MgO: magnesium oxide
ESMS: electrospray mass spectrometry
ICP: inductively coupled plasma
NMR: nuclear magnetic resonance
HSQC: heteronuclear single quantum coherence
HMBC: heteronuclear multiple bond correlation
FT-IR: Fourier transform infrared radiation
TGA: thermogravimetric analysis
DSC: differential scanning calorimetry
