# Supplementary material for: Synthesis, Characterization, and Cellular Uptake of a Glycylglycine Chelate of Magnesium
Source: ACS Omega. 2021 Nov 30;6(49):33454–61. doi: 10.1021/acsomega.1c04146 (PMC8679001; doi:10.1021/acsomega.1c04146)
Supplement: Supplementary file 1 — ao1c04146_si_001.pdf [file ao1c04146_si_001.pdf]

Supplemental Information

Synthesis, Characterization, and Cellular uptake of a Glycylglycine Chelate of Magnesium

Derek R. Case,[†] Ren Gonzalez,[‡] Jon Zubieta,[†] Robert P. Doyle[†,*]

[†]111 College Place, Department of Chemistry, Syracuse University, Syracuse, New York, 13244, USA.

[‡] Balchem Corporation, 52 Sunrise Park Road, New Hampton, NY 10958, USA.

[*] Correspondence: rpdoyle@syr.edu

Table of Contents

ESI Mass Spectrum of **1** (**S1**) Page 2

Full Spectra ^{1}H NMR of Glycylglycine (**S2**) Page 3

^{1}H NMR of **1** Full Spectra (**S3**) Page 4

^{1}H NMR Overlay of Glycylglycine and **1** (**S4**) Page 5

^{1}H NMR Overlay of Glycylglycine, **1**, Citric Acid and Magnesium Citrate (**S5**) Page 6

^{13}C NMR of Glycylglycine (**S6**) Page 7

^{13}C NMR Overlay of Glycylglycine and **1** (**S7**) Page 8

^{13}C NMR Overlay of Glycylglycine, **1**, and Magnesium Citrate (**S8**) Page 9

Full Spectra HSQC of Glycylglycine and **1** (**S9**) Page 10

Full Spectra HMBC of Glycylglycine and **1** (**S10**) Page 11

Elemental Analysis and ICP of **1** (**S11**) Page 12

Linear Regression of Magnesium Uptake Standard (**S12**) Page 13

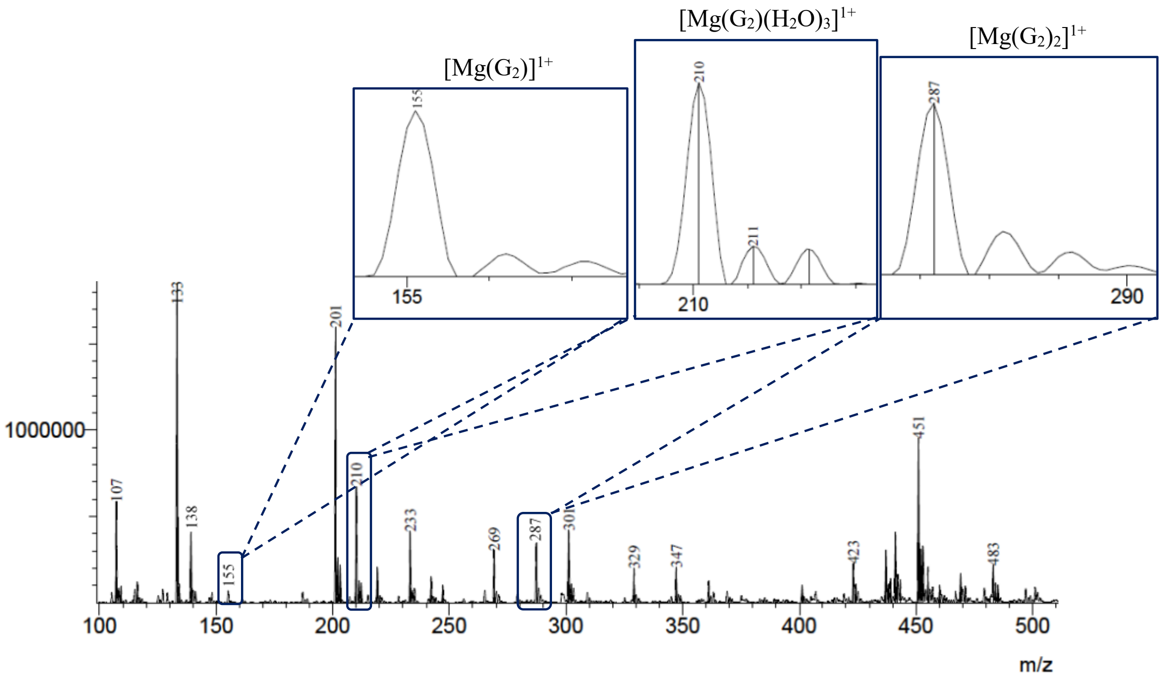

Figure S1. ESI-MS mass spectrum of **1** exhibiting an appropriate magnesium isotopic distribution pattern (IDP) and the presence of **1** along with an *in situ* formed 1:2 complex.

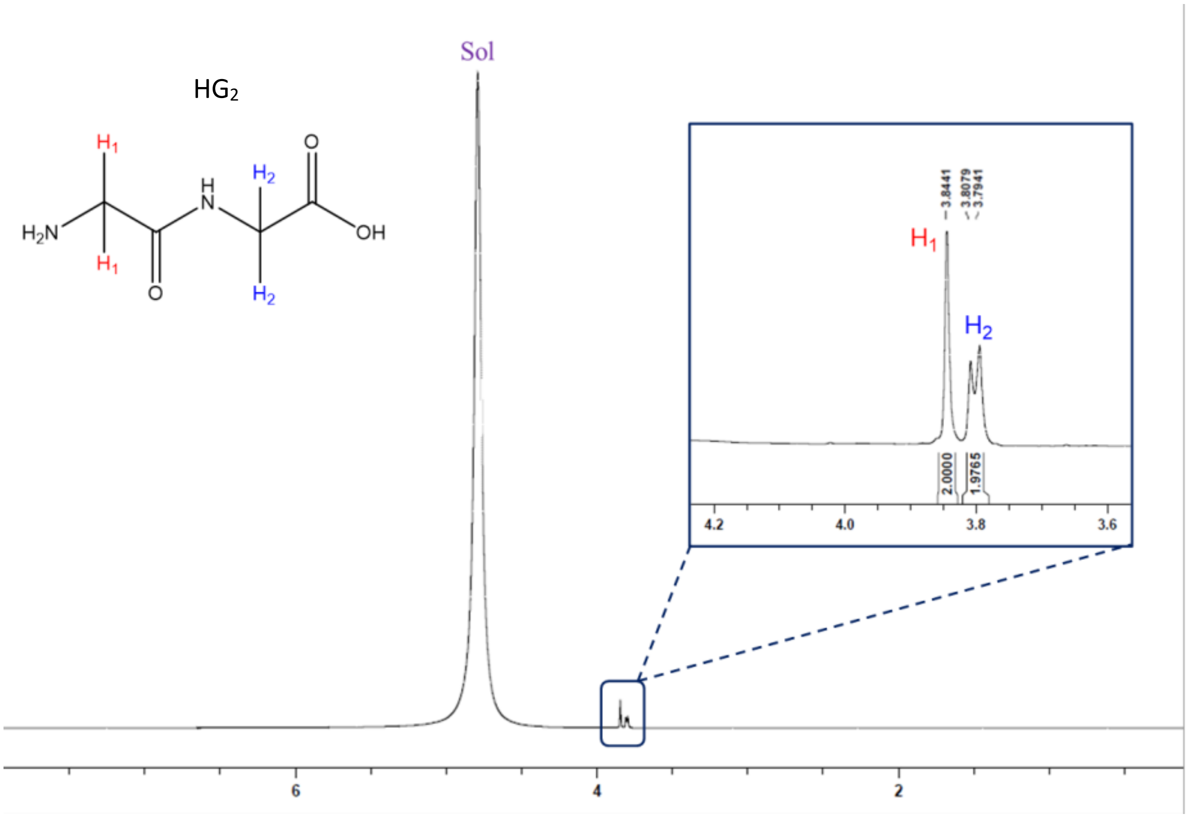

*NMR taken in D_2O

Figure S2. The full spectrum ^{1}H NMR of HG_2 showing a clean singlet and a signal with observed coupling – the observed integration coincides with the expected integration.

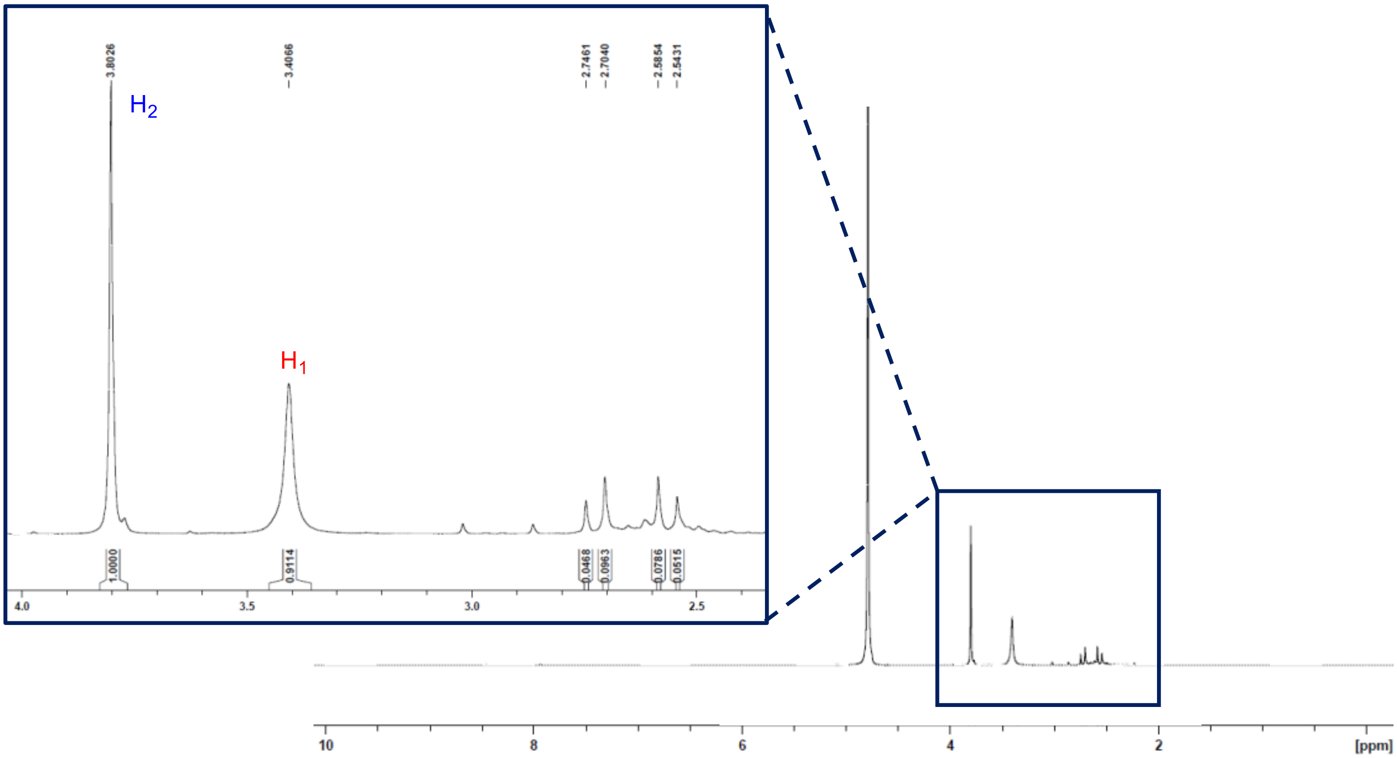

*NMR taken in D_2O

Figure S3. Full spectrum ^{1}H NMR of **1** showing two singlets and the presence of magnesium citrate. The observed integration coincides with the expected integration values.

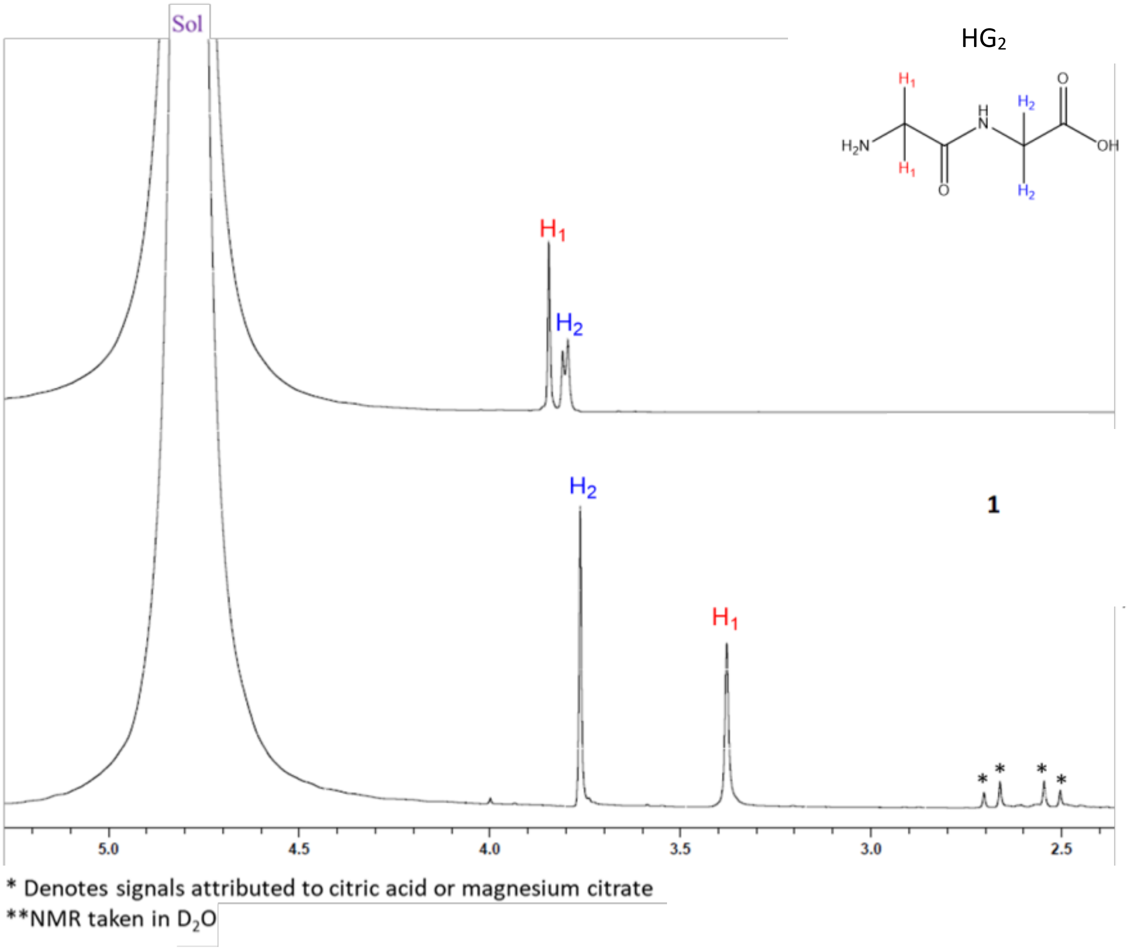

Figure S4. ^{1}H NMR spectra overlay of HG_2 (**Top**) and the 1 complex (**Bottom**) – the ^{1}H NMR spectrum of **1** shows the presence of magnesium citrate, but the desired integration is conserved between HG_2 and**1**. Observed coupling of the H_2 proton of HG_2 is no longer observed for **1**.

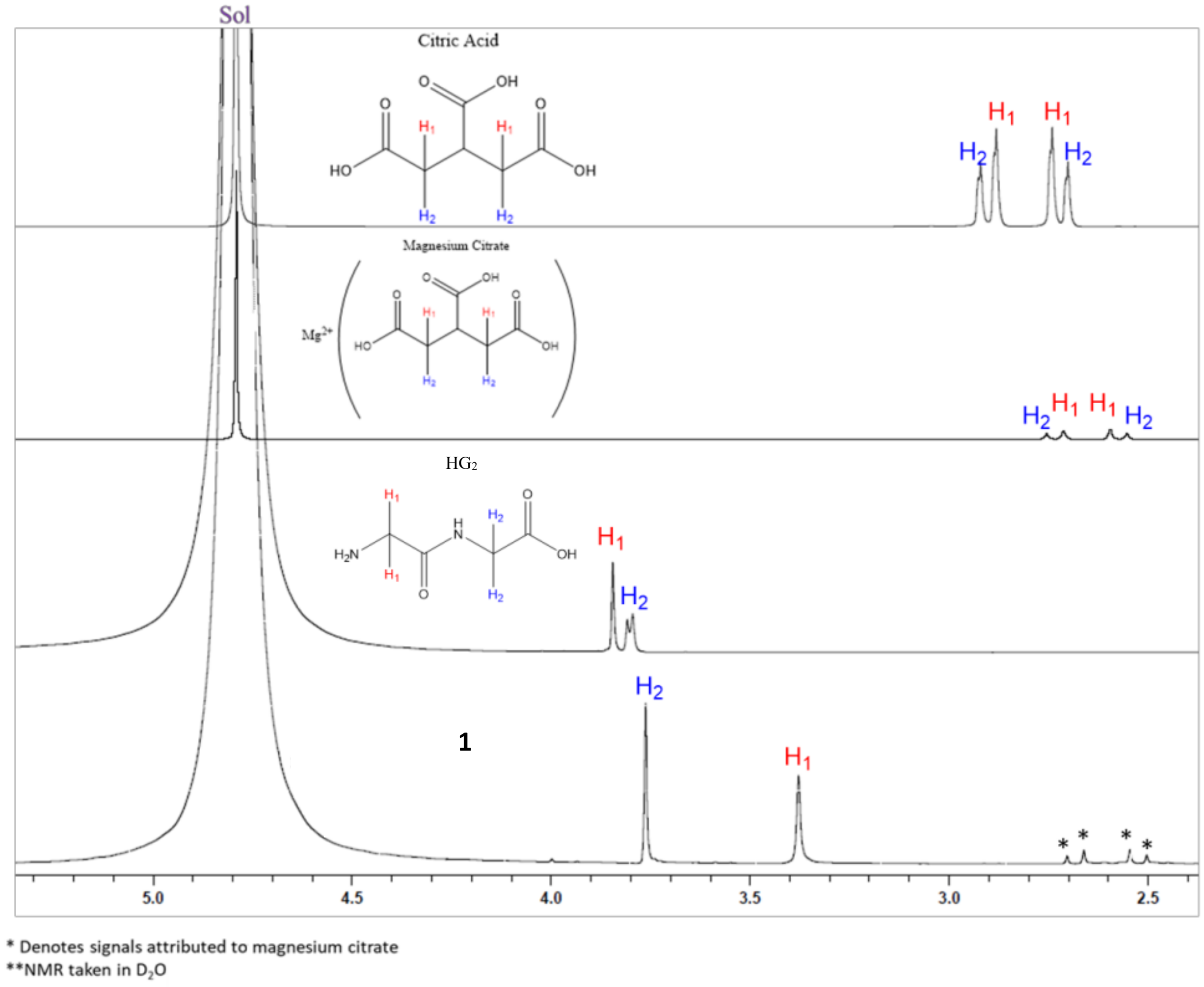

Figure S5. ^{1}H NMR spectra overlay for citric acid (**Top**), magnesium citrate (**Second from Top**), HG_2 (**Third from Top**), and **1**(**Bottom**). The integration is conserved between HG_2 and **1**, consistent with the formation of magnesium citrate.

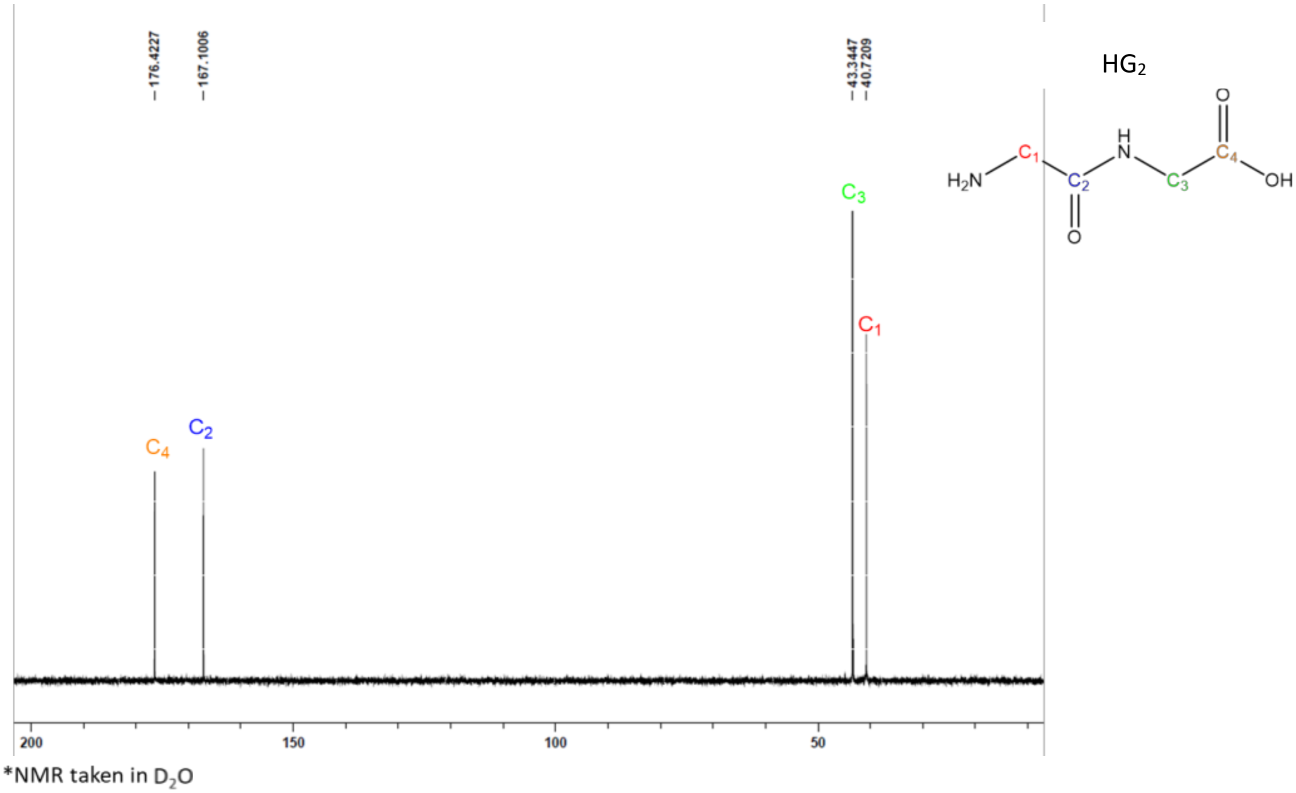

Figure S6. Full spectrum ^{13}C NMR of HG_2 .

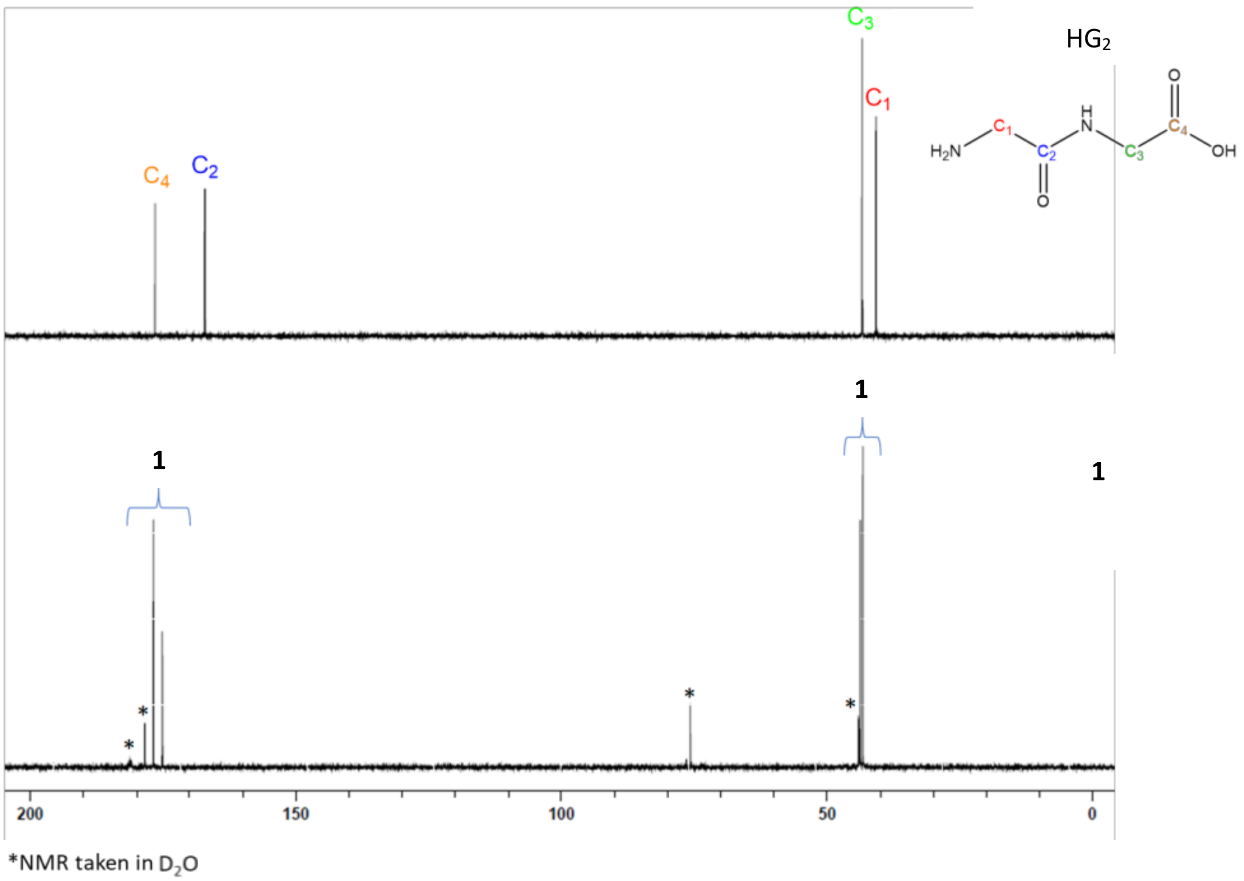

Figure S7. Full spectra ^{13}C NMR overlay of HG_2 (**Top**) and **1** (**Bottom**). **1** showed four signals attributed to magnesium citrate as marked by the asterisks, but the observation of four carbon signals attributed to HG_2 are conserved.

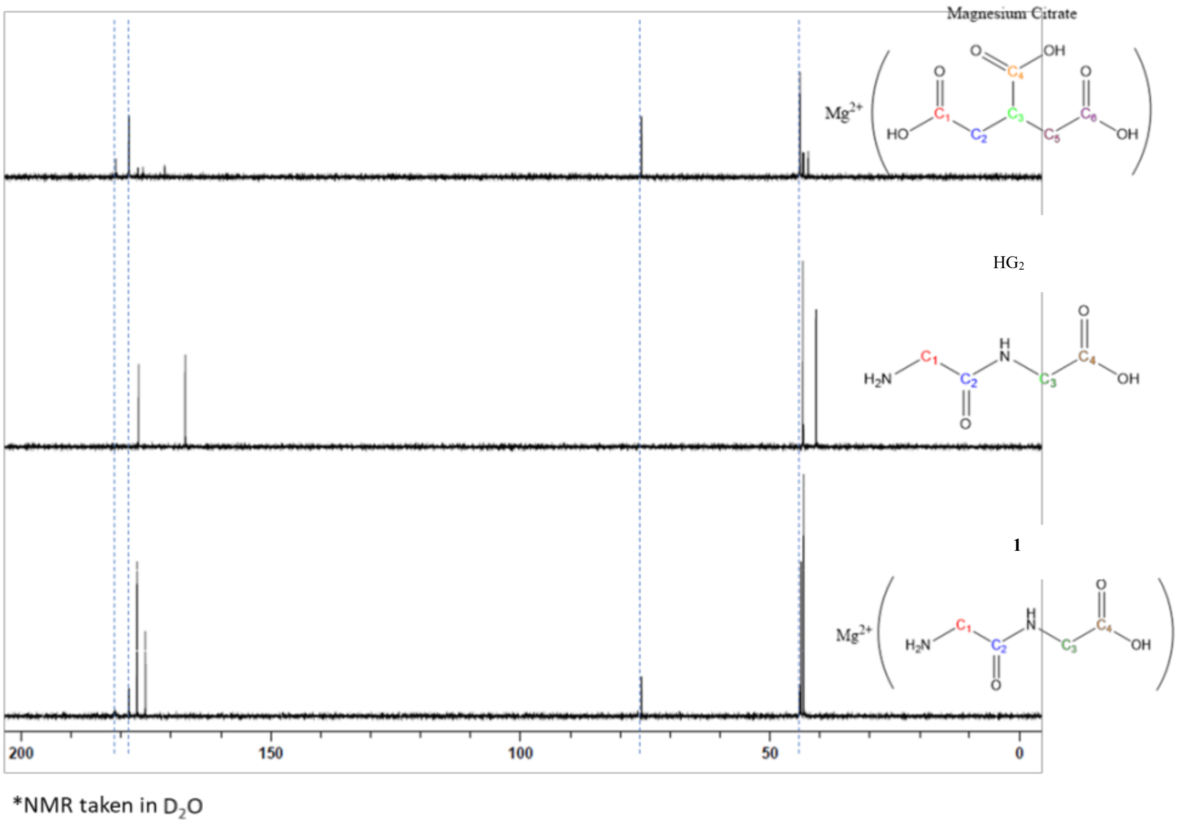

Figure S8. Full spectra ^{13}C NMR overlay of magnesium citrate (**Top**), HG_2 (**Middle**), and **1** (**Bottom**). The overlay illustrates the impurity observed in the spectra of **1** attributed to magnesium citrate, while the carbon signals attributed to HG_2 are conserved.

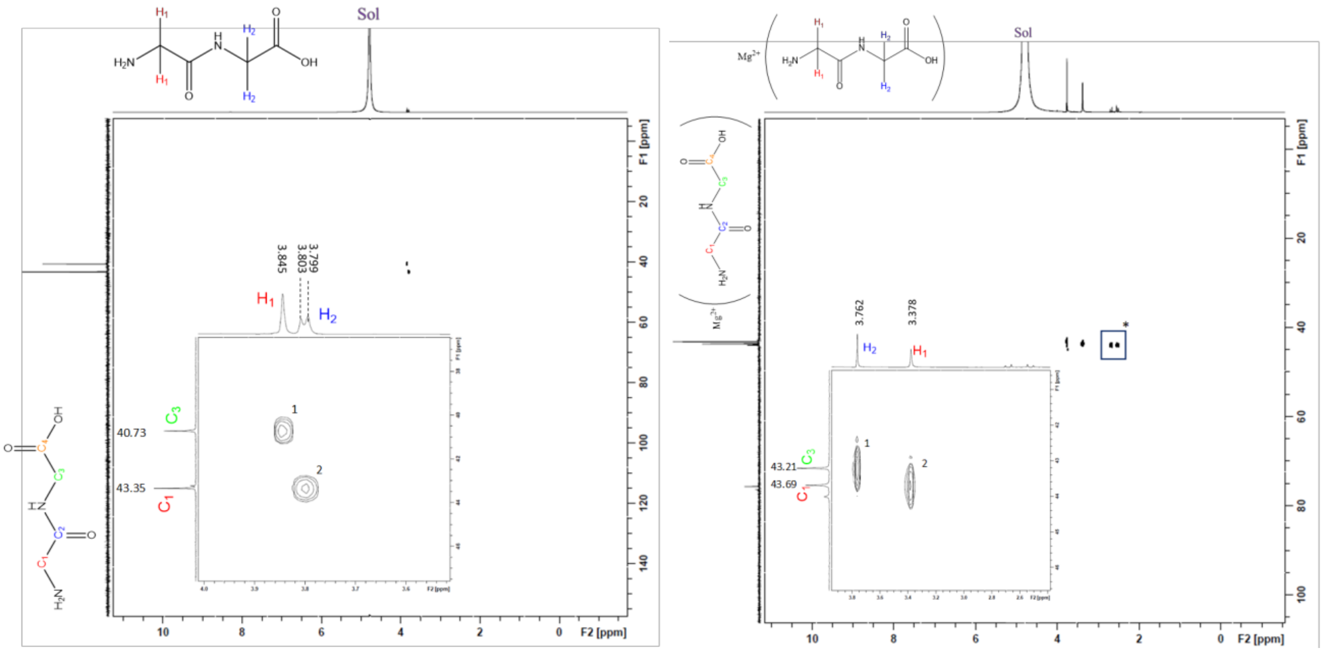

* Denotes signals attributed to magnesium citrate

**NMR taken in D_2O

Figure S9. Full spectra HSQC of HG_2 (**Left**) and HSQC of **1** (**Right**) utilized to confirm the proton and carbon assignments of HG_2 and **1**.

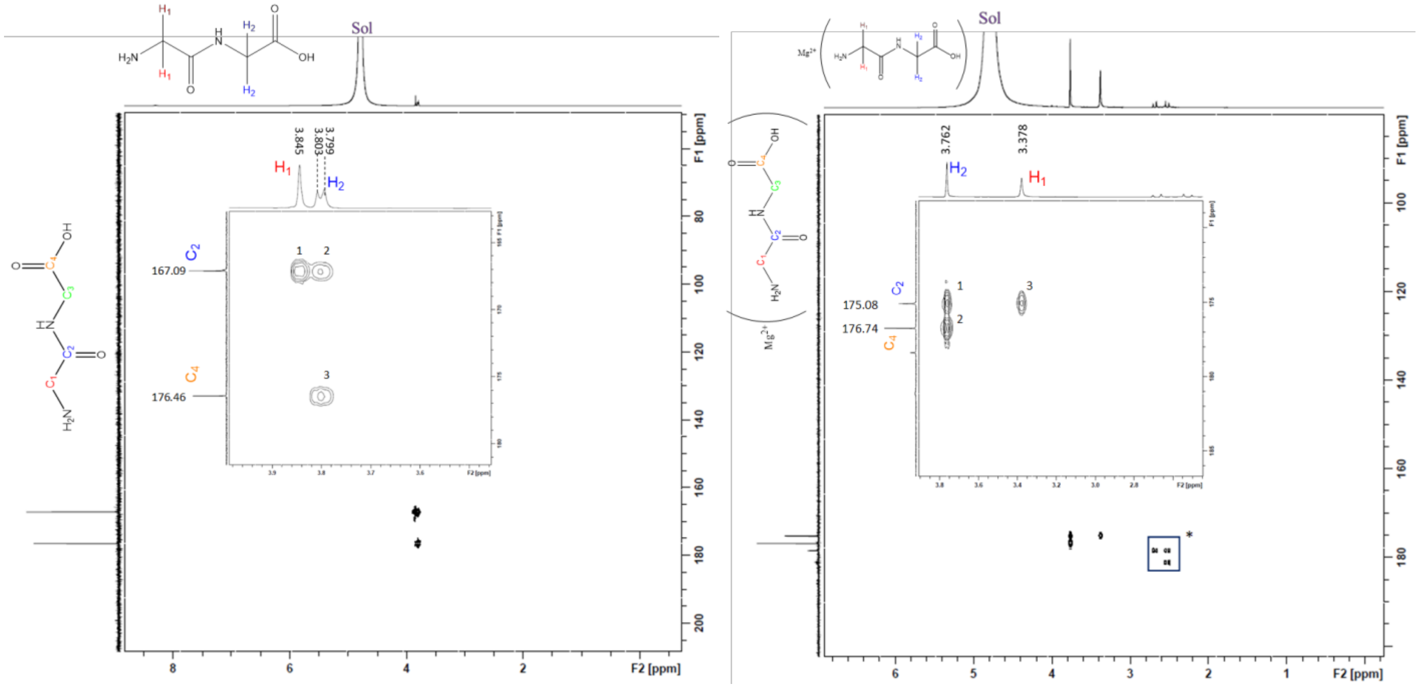

* Denotes signals attributed to magnesium citrate
**NMR taken in D_2O

Figure S10. Full spectra HMBC of HG_2 (**Left**) and HMBC (**Right**) of **1** utilized to confirm the proton and carbon assignments of HG_2 and **1**.

Analytical Report
Report Number: 185635
Report Status: *Final*

Mg	N
8.78 %	9.85 %
	9.92 %

Figure S11. ICP analysis of **1**.

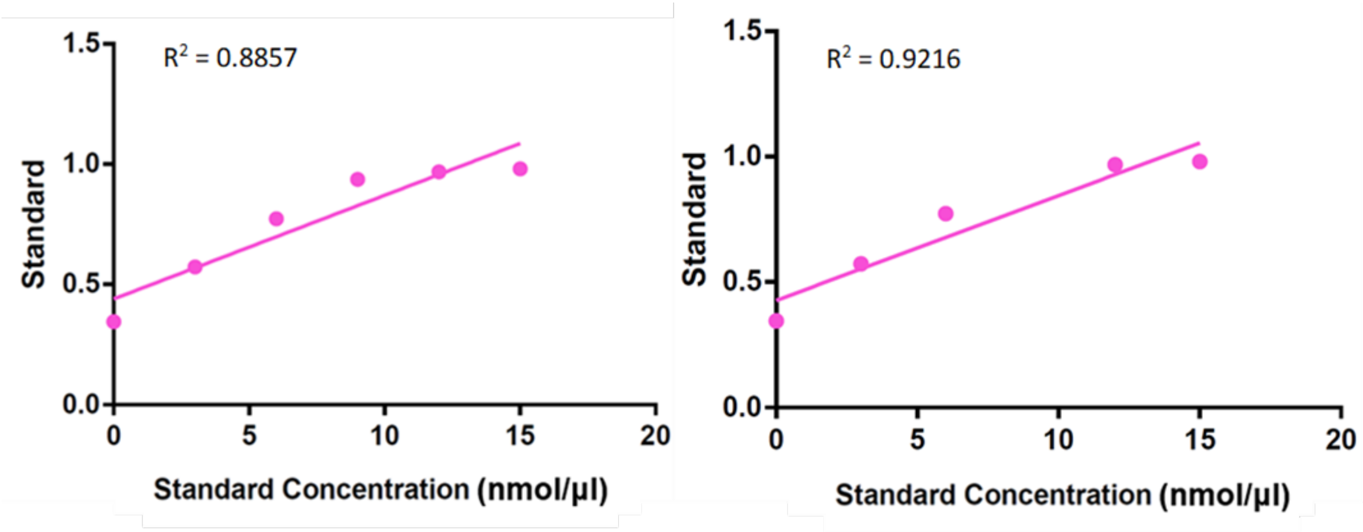

Figure S12. Plot of the linear regression of magnesium assay kit standard to confirm linearity.
